# Differential expression of proteins and phosphoproteins during larval metamorphosis of the polychaete *Capitella *sp. I

**DOI:** 10.1186/1477-5956-9-51

**Published:** 2011-09-03

**Authors:** Kondethimmanahalli H Chandramouli, Lisa Soo, Pei-Yuan Qian

**Affiliations:** 1 KAUST Global Collaborative Research, Division of Life Science, Hong Kong University of Science and Technology, Hong Kong SAR, China

**Keywords:** *Capitella *sp. I, larval metamorphosis, multiplexed proteomics, 2-DE, phosphoproteome, RT-PCR

## Abstract

**Background:**

The spontaneous metamorphosis of the polychaete *Capitella *sp. I larvae into juveniles requires minor morphological changes, including segment formation, body elongation, and loss of cilia. In this study, we investigated changes in the expression patterns of both proteins and phosphoproteins during the transition from larvae to juveniles in this species. We used two-dimensional gel electrophoresis (2-DE) followed by multiplex fluorescent staining and MALDI-TOF mass spectrometry analysis to identify the differentially expressed proteins as well as the protein and phosphoprotein profiles of both competent larvae and juveniles.

**Results:**

Twenty-three differentially expressed proteins were identified in the two developmental stages. Expression patterns of two of those proteins were examined at the protein level by Western blot analysis while seven were further studied at the mRNA level by real-time PCR. Results showed that proteins related to cell division, cell migration, energy storage and oxidative stress were plentifully expressed in the competent larvae; in contrast, proteins involved in oxidative metabolism and transcriptional regulation were abundantly expressed in the juveniles.

**Conclusion:**

It is likely that these differentially expressed proteins are involved in regulating the larval metamorphosis process and can be used as protein markers for studying molecular mechanisms associated with larval metamorphosis in polychaetes.

## 1. Background

The polychaete *Capitella sp*. I is a widely distributed marine benthic worm. It is considered to be the most opportunistic and pollutant-tolerant species of benthic marine invertebrate [1]. This species has been widely used as a biomonitor of pollutants in marine environments. It is also currently being developed as a model for developmental studies [2]. Similar to most benthic polychaetes, this worm has a biphasic life cycle during which larvae settle on soft sediments and spontaneously metamorphose into benthic juveniles [3]. *Capitella sp*. I undergoes semi-direct development, generating approximately a dozen segments during the larval stage [4]. After hatching and release from brood tubes, non-feeding, pelagic larvae can undergo metamorphosis within hours in response to chemical settlement cues. Metamorphosis results in the transition to a benthic lifestyle with only minor morphological changes, including elongation of the body, loss of cilia needed for swimming, and development of capillary setae and hooded hooks necessary for crawling through sediments [5-7]. A variety of studies on recruitment and population dynamics [8], settlement induction [5], the segmentation process [9], molecular-level signaling mechanisms [10], and gene expression [2] during larval metamorphosis have been conducted on this ubiquitous marine worm. That said, no study has been published on proteomic changes associated with larval metamorphosis in *Capitella *sp. I despite rapid developments in proteomics technologies and their application to understanding complex larval metamorphic processes [11,12].

Our previous studies demonstrated that larval development and metamorphosis in the polycheates *Pseudopolydora vexillosa *[13] and *Hydroides elegans *[14] were mediated by changes in both protein expression and phosphorylation status. In competent *P. vexillosa *larvae, calreticulin, tyrosin 3-monooxygenase activation protein, and the cellular matrix were up-regulated [13], whereas most of the larval proteins identified in *H. elegans *were isoforms of tubulin, suggesting the probable association between microtubule dynamics and larval development [14]. It has been argued that the specific mechanisms of larval development and metamorphosis vary from species to species [15,16] because the metamorphic transitions in different species likely evolved under different selective pressures [16]. For example, an *H. elegans *larva undergoes rapid and substantial tissue remodulation during metamorphosis [17,18] and becomes a tube-dwelling juvenile with a branchial crown, whereas a *Capitella *sp. I larva metamorphoses spontaneously and requires little tissue remodulation resulting in minor morphological changes [7]. We hypothesized that the protein expression pattern during larval settlement and metamorphosis in the polychaete *Capitella *sp. I differs from that in the polycheates *P.vexillosa *[13] and *H.elegans *[14]. To test this hypothesis, we analyzed the proteome of competent larvae and juveniles of *Capitella *sp. I to identify differentially expressed proteins and then we made comparisons among the three polychaete and non-polychaete species.

## 2.0. Results

### 2.1. Mapping proteins and phosphorylated proteins during larval metamorphosis in *Capitella *sp. I

**Figure 1:**
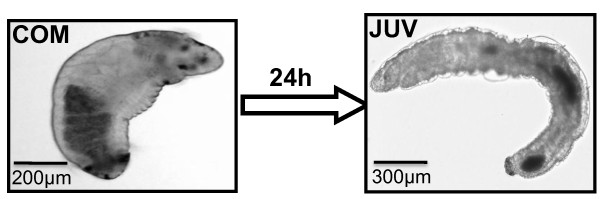
**Developmental stages of the polychaete annelid *Capitella sp. I***. Two developmental stages were chosen for proteomic analysis: (A) competent larva and (B) juvenile.

**Figure 2:**
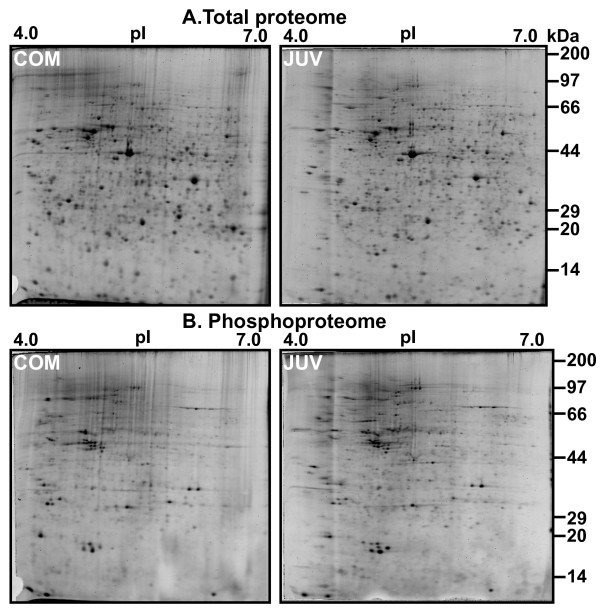
**Representative 2-DE gel images of competent larva and juvenile of *Capitella sp*. I**. 300 μg of total protein extracts were separated on linear pH 4-7 IPG strips followed by 12.5% polyacrylamide gel electrophoresis. Upper panel A: 2-D gels stained for total proteome with Sypro Ruby fluorescent dye. Lower Panel B: 2-D gels stained for phosphoproteome with ProQ diamond fluorescent dye.

**Figure 3:**
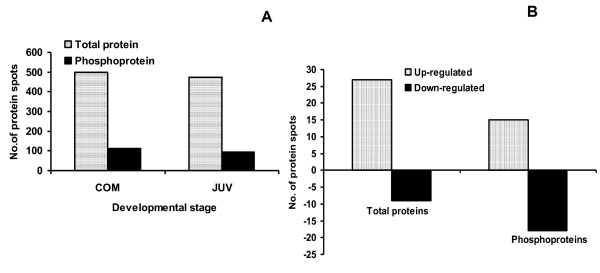
**Differential analysis of protein spots**. (A) The number of protein and phosphoprotein spots reproducibly detected in competent (COM) and Juvenile (JUV) stages, (B) The number of differentially expressed total protein spots and phosphoprotein spots in competent (COM) and Juvenile (JUV) stages. Differentially expressed spots showed significant differences between two stages (Student's *t*-test (*p *< 0.01, n = 3).

**Figure 4:**
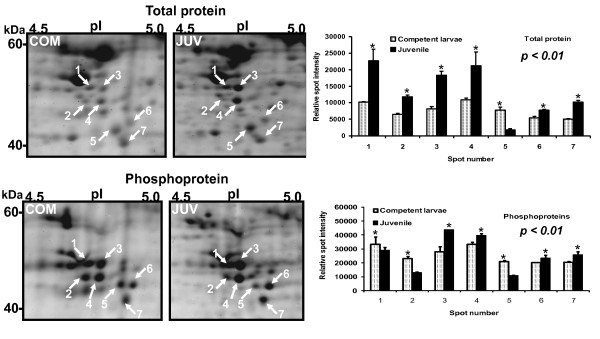
**Differentially expressed, low-abundant phosphoproteins (spots 1-7) in competent and juveniles stages of *Capitella sp*. I**. A close view of total proteins and phosphoproteins and their relative spot intensity

**Figure 5:**
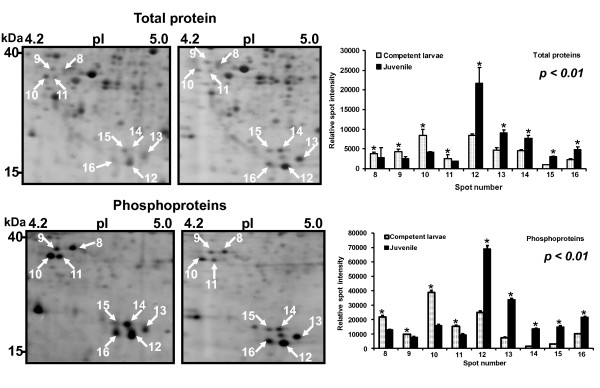
**Differentially expressed, low-abundant phosphoproteins (spots 8-16) in competent and juveniles stages of *Capitella sp*. I**. A close view of total proteins and phosphoproteins and their relative spot intensity

Representative 2-DE gels of sequentially stained phosphoproteins and total proteins in the two developmental stages (Figure 1) of competent larvae (COM) and juveniles (JUV) of *Capitella *sp. I are shown in Figure 2. Protein spots that exhibited a 1.5-fold increase or decrease in spot intensity in the results of either of the two staining methods used in this study were selected for further analyses. In the COM and JUV stages, 498 and 473 protein spots and 113 and 94 phosphoprotein spots (Figure 3A) were detected, respectively. Of these, 27 protein spots and 15 phosphoprotein spots were up-regulated (>1.5-fold) and 9 protein spots and 18 phosphoprotein spots were down-regulated (<1.5-fold) during the transition period from competent larvae to juveniles (Student's *t*-test, *p *< 0.01) (Figure 3B). Most of the phosphorylated proteins were present in low concentrations and were under the detection limit of the CCB stain. To trace the changes in the expression levels of these phosphorylated proteins, selected areas of the phosphoproteome gels were enlarged and 16 spots with low abundance were found to be differentially expressed in the COM and JUV. Nine phosphoproteins spots (Spot Nos. 3, 4, 6, 7 and 12-16) were up-regulated and 7 phosphoprotein spots (Spot Nos. 1, 2, 5 and 8-11) were down-regulated in the JUV (Figure 4 and 5; lower panel). A similar trend of differential expression of total proteins was observed when the gels were stained with Sypro Ruby dye (Figures 4 and 5; upper panels). A comparison of the intensity of the total protein spots with that of the phosphoprotein spots revealed that changes in the expression of the proteins were accompanied by changes in phosphorylation levels. On the other hand, changes in phosphoprotein expression were merely due to differences in the total protein expression.

### 2.2. Identification of differentially expressed proteins by MALDI-TOF/MS

**Figure 6:**
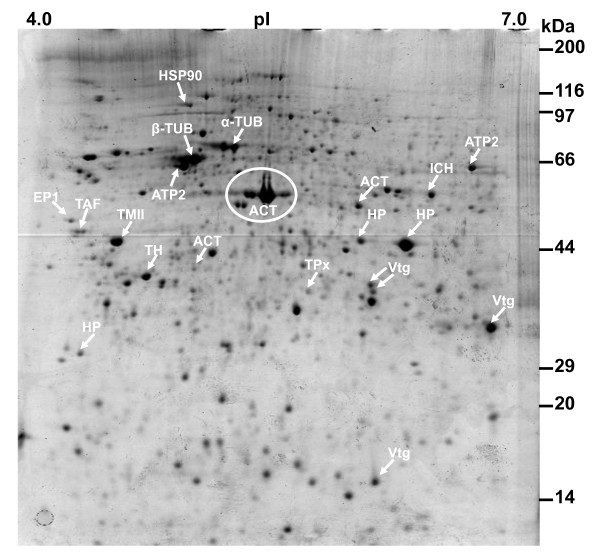
**2-D gel of competent larvae of *Capitella sp*. I stained with modified G-250 Colloidal Coomassie Blue**. The protein spots (marked with an arrow and circle) were identified by MALDI-TOF/TOF.

**Figure 7:**
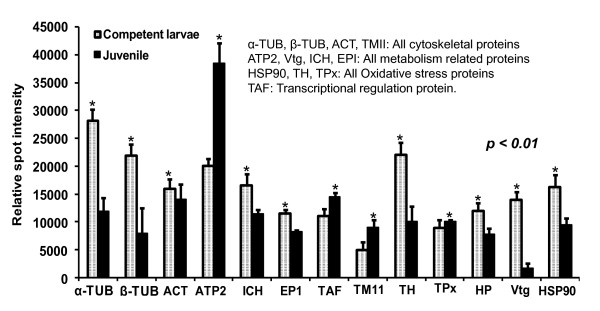
**Relative expression levels of identified proteins in competent larvae and juveniles during metamorphosis in *Capitella sp*. I**. The relative intensity of protein spots was determined using the PD Quest software.

Twenty-three proteins were identified and are listed in Table 1. The observed MW and p*I *values of the identified proteins were very close to the theoretical values derived from a search in the *Capitella *sp.I genome database and the Swiss-Prot database. Many of the proteins identified in the *Capitella *genome database were also identified in the Swiss-Prot database with high confidence scores, suggesting the high accuracy of protein identification. Notably, several protein spots were identified as the same protein or isoforms of the same protein, such as tubulins (TUB) (spots 17 and 18); actins (ACT) (spots 20, 21, 22 and 30); vitellogenins (Vtg) (spots 33, 34, 37, and 38); ATP synthase (ATP2) (spots 19 and 23) as shown in Figure 6 and listed in Table 1. Cytoskeleton proteins accounted for 33% of the identified spots, possibly due to the prevalence of different isoforms caused by protein modification or protein degradation during larval metamorphosis. The cytoskeletal proteins and Vtg were down-regulated in our samples as shown in Figure 7. Tropomyosin (TM 11, spot 28) was up-regulated in the JUV. Three identified proteins were involved in protein metabolism pathways in mitochondria: (i) Isocitrate dehydrogenase-2 (ICH, spot 24), (ii) Enolase-phosphatase (EP1, spot 26) and (iii) ATP2. ICH and EP1 were down-regulated and ATP2 was up-regulated in the JUV (Figure 7). Another three proteins that were identified may play a role as a defense mechanism and in oxidative stress: (i) Tyrosine 3-monooxygenase (TH, spot 29), (ii) Thioredoxin peroxidase (TPx, spot 35), and (iii) Heat shock protein 90 (HSP90, spot 39). TH and HSP90 were down-regulated and TPx slightly increased in the JUV. Template activating factor (TAP, spot 27) possibly involved in transcriptional regulation was also identified. It was up-regulated in the JUV. Furthermore, some novel proteins differentially expressed during metamorphosis were identified as hypothetical proteins (HP) (Spots 31, 32, and 36) (Table 1 and Figure 6). There was no significant protein match for these proteins in the *Capitella sp *I and Swiss-Prot databases.

**Table 1:** Identification of abundant proteins in *Capitella Sp *I during metamorphosis by MALDI-TOF/TOF.

**Spot No**.	Acc No.^a)^	Protein name ^b)^	MW(kDa)^c)^ **Obs./Theo**.	pI^c)^ **Obs./Theo**.	PM/SC (%)	Fold Change (Total protein)	Fold Change (mRNA)
17	163388/P68370	Alpha -tubulin (α-TUB)	40/50	5.6/5.5	35/11	-2.4	-1.1

18	156132/P11833	Beta-tubulin ((β-TUB)	43/45	5.7/5.3	20/6	-2.7	NA

19	157862/Q9PTY0	ATP synthase beta subunit (ATP2)	46/44	5.0/5.3	33/9	+1.9	+ 2.2

20	158679/P68142	Actin cytoplasmic type 8 (ACT)	42/42	5.3/5.6	16/4	-1.1	+ 1.6*

21	158679/P68142	Actin cytoplasmic type 2 (ACT)	59/40	5.6/5.5	16/9	-1.1	+ 1.6*

22	158679/P68142	Beta-actin(ACT)	39/40	5.7/5.6	34/12	-1.1	+ 1.6*

23	158616/Q5R546	ATP synthase alpha subunit (ATP2)	59/56	8.5/7.0	38/23	+1.9	+ 2.2

24	163979/P48735	Isocitrate dehydrogenase-2 (ICH)	44/48	5.9/6.4	27/9	-1.5	NA

25	160805/P79818	Cytoplasmic beta actin (ACT)	40/40	5.6/6.0	25/7	-1.1	NA

26	226906/Q6GM17	Enolase-phosphatase E1 (EP1)	21/40	4.1/4.3	27/5	-1.5	NA

27	153159/Q01105	Template activating factor (TAF)	30/35	4.1/4.3	26/5	+1.5	+ 2.5

28	156731/P43689	Tropomyosin-2(TMII)	33/33	4.6/4.5	40/12	+1.8	NA

29	152326/P62258	Tyrosine 3-monooxygenase (TH)	29/32	4.7/4.7	25/8	-2.2	-1.1

30	158679/P68142	Actin cytoplasmic type 1 (ACT)	41/35	5.5/5.3	31/8	-1.1	+ 1.6*

31	221075	Conserved hypothetical protein (HP)	52/43	7.8/6.2	15/7	NA	NA

32	221075	Conserved hypothetical protein (HP)	52/40	7.8/6.5	31/16	NA	NA

33	209306/P55155	Vitellogenin (Vtg)	176/35	7.1/6.3	8/10	-8.0	-2.0*

34	209306/P55155	Vitellogenin (Vtg)	176/33	7.1/6.3	11/13	-8.0	-2.0*

35	180369/Q63716	Thioredoxin peroxidase (TPx)	26/28	6.7/6.2	41/8	+1.1	-1.4

36	175529/Q23280	hypothetical protein (HP)	23/29	4.3/4.3	11/3	NA	NA

37	209306/P05690	Vitellogenin (Vtg)	176/18	7.1/7.0	11/13	-3.0	-2.0*

38	198200/P05690	Vitellogenin (Vtg)	174/14	7.9/5.5	3/7	-2.0	-2.0*

39	159573/P07900	Heat shock protein-90 (HSP90)	95/45	4.8/5.0	7/18	-1.8	NA

a) Accession numbers are from the JGI *Capitella *genome database and the Swiss-Prot database b) For positive identification, the score had to be over the significance threshold (*p*<0.05). c) Observed (Obs.) MW and p*I *values were estimated from 2-DE gels and Theoretical (Theo.) MW and p*I *values were derived from a database search by MASCOT. PM: number of peptides matching the protein sequence; SC: sequence coverage; *: Total mRNA expression levels; NA: Not determined.

### 2.3. Analysis of differential expression of proteins on the translation and gene expression level

**Figure 8:**
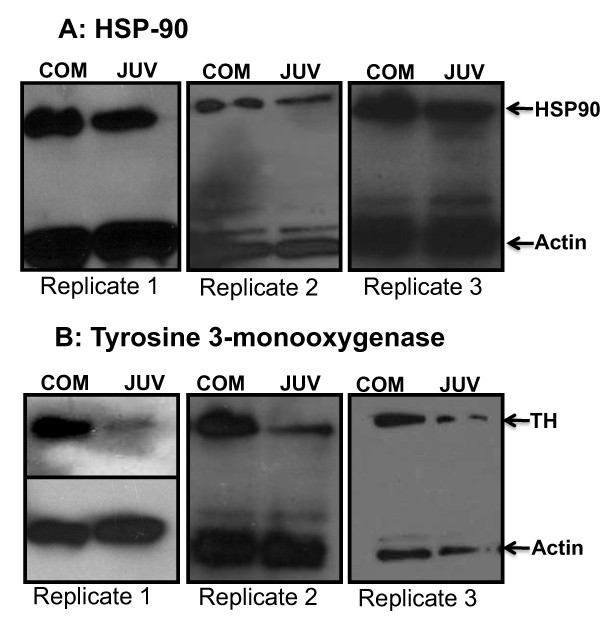
**Western blot analysis**. 20 μg of protein was separated on 10% SDS-PAGE gel. The membranes were incubated with anti-HSP90 (A) and anti tyrosine 3-monooxygenase (B) monoclonal antibodies and developed by the ECL Western blotting analysis system.

Western blot analysis was performed to confirm the expression levels of selected differentially expressed proteins identified by the proteomic approach. Because antibodies against *Capitella *sp. I proteins were not commercially available, only two identified proteins, HSP90 and TH, were adopted for analysis in the COM and JUV stages. Consistent with the 2-DE results, the expression levels of the proteins decreased during the transition from competent larvae to juveniles as shown in Figure 8.

**Figure 9:**
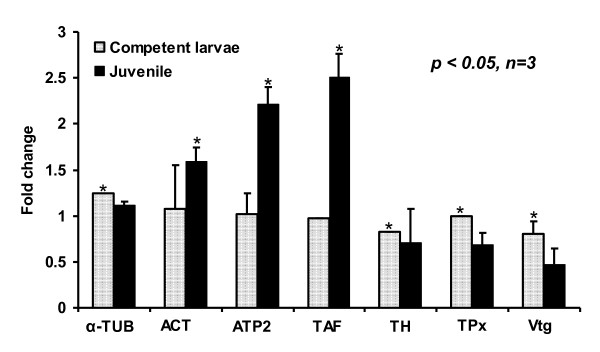
**The gene expression profile of proteins in the competent larva and juvenile stages of *Capitella sp*. I**. Total RNA was isolated from the COM and JUV. The 18S gene was used for normalization of the compared templates. The values are mean ± standard deviation obtained by normalization of target genes against 18S (significant difference is compared to competent larvae by a Student's *t-*test, *p *< 0.05).

Seven genes encoding the identified proteins listed in Table 2 were analyzed using semi-quantitative RT-PCR. TUB, TH, and Vtg showed decreased transcriptional expression during metamorphosis while ATP2 and TAF showed increased expression (Figure 9), consistent with the 2-DE results (Figure 7). ACT, the cytoskeleton protein, experienced an increase in transcription, but it did not experience a significant increase in the protein expression level. The oxidative stress protein, TPx, had a typical increase in gene expression levels during metamorphosis, which differed from the proteomics data, indicating a constant protein level. The reason for this difference might be post-transcriptional regulation or differences in mRNA and protein turnover rates. Of the seven genes tested, five had a positive correlation between the transcriptional and translational expression profiles, whereas two genes followed a reversed expression trend.

**Table 2:** Gene-specific primers used for real-time PCR

Name of the gene	**Accession no**.	Primer sequence
Template activating factor 1	153159	FP: 5'-GGCCCAACCCCCTCCAGTAC-3' RP: 5'-AGCCAGAATGGGCTGTGGTGC-3'

Actin, cytoplasmic	158679	FP: 5'-ACGAAGTTGCCGCTCTTGTCATC-3' RP: 5'-GCCCATACCGACCATGACACCC-3'

ATP synthase beta subunit	157862	FP: 5'-CCGCCACTCCCAAGGGCAAA-3' RP: 5'-CGCACTCCTGGCCACGGATC-3'

Thirodoxin peroxidase	180369	FP: 5'-TGCGCCAGGTGACCATCAACG-3' RP: 5'-GCAGCACCTCATCGACCGACC-3'

Alpha tubulin	163388	FP: 5'-CACGTCCCCCGTGCCGTAA-3' RP: 5'-CCAGTGCGGACCTCATCAACC-3'

Vitellogenin	209306	FP: 5'-GTCCGCGCAGCGCTCAAATG-3' RP: 5'-GGAGCTGGCGGAACGAAGCA-3'

Tyrosine monooxygenase	152326	FP: 5'-CGGGCGCAGCCATCTTGATTG-3' RP: 5'-ACGCGACGGACAGCAGGTTTC-3'

18s RNA (Reference gene)	AF508118	FP: 5'-GGAAAACTCACCCGGCCCGG-3' RP: 5'-CGACCCGCAGAACGGATCGG-3'

## 3.0. Discussion

Previous studies elucidating molecular mechanisms during larval metamorphosis in *Capitella Sp*. I focused on the influence of hormone-mediated chemical signals that are mediated by protein kinase C (PKC) and ion channels and components of the Notch signaling pathway [6.10]. The chemical cues present in the ocean environment can trigger larval metamorphosis in polychaete species and the settlement signals appear to be species specific [19]. Biggers and Laufer reported that PKC activation causes several cellular events that transduce juveniles to mediate the settlement and metamorphosis of the *Capitella *larvae. Moreover, PKC activation transduces the external juvenile hormone (JH) signal and leads to subsequent modulation of ion channels. PKC activation by JH may activate transcription factors, such as nuclear factor-kB, or it may stimulate the mitogen-activated protein kinase pathway and thereby trigger settlement and metamorphosis [6]. Thamm and Seaver provided evidence of coordinated gene expression of some members of the Notch signaling pathway and Notch-independent activation in *Capitella sp. I*. Spatial localization of Notch signaling components correlates with areas of high cell proliferation during *Capitella *sp. I development [10].

### Comparison of proteomes and phosphoproteome profiles of *Capitella *sp. I with polycheates *Pseudopolydora vexillosa *and *Hydroides elegans*

Larval metamorphosis of *Capitella *sp. I requires minor morphological changes, such as body elongation and loss of larval structures, which may be mediated by post-translational modifications and protein degradation. The reduction in the number of proteins (25 spots) and phosphoproteins (19 spots) in juveniles (Figure 3A) could be related to the loss of larval cilia and protein degradation given that those larval structures are no longer needed by metamorphosed juveniles. Our previous studies revealed that *P. vexillosa *and *H. elegans *experience a drastic decrease in the number of total protein spots (>200 spots) during metamorphosis [13,14]. These intra-specific differences can be attributed to differences in the developmental process and consequential morphological changes among these three polychaete species. For instance, *Capitella *sp. I experiences less structural loss and minor morphological changes during metamorphosis and does not require substantial development of juvenile organs [7]. In contrast, larval metamorphosis of *H. elegans *requires substantial tissue reorganization and drastic morphological changes during metamorphosis, whereas the competent larvae of *P. vexillosa *constantly accumulate and differentiate adult features and feeding structures when they undergo transient structural reorganizations during metamorphosis [3]. The up-regulation of the total proteins (27 spots) and phosphoproteins (7 spots) in the COM larvae of *Capitella *sp. I may be due to synthesis and accumulation of large amounts of proteins that are required for the elongation of the body and the development of the capillary setae during competency. Our previous work revealed that the early phase of metamorphosis in polychaetes does not require *de novo *transcription and translation. We suspect that most of the up-regulated proteins are synthesized and stored in the competent larvae while attaining competency. In contrast, we observed a drastic down-regulation of total proteins (>150 spots) from the competent larval stage to adults in *H. elegans*. This drastic change in the number of protein spots accrued during metamorphosis may be associated with segmentation processes in polychaete species. In both *Capitella *sp. I and *H. elegans*, once the segments become morphologically visible, there is a dramatic decrease in the number of dividing cells in the mid-body region. This change occurs quite abruptly in *H. elegans *and is coincident with segment formation. In *Capitella *sp. I, it is a gradual process that occurs over several days [9]. Furthermore, the competent larvae of *H. elegans *and *P. vexillosa *had more stage-specific total protein and phosphoprotein spots when compared to the COM stage of *Capitella *sp. I. This observation suggests that the morphological changes during larval metamorphosis in *Capitella sp. I *are not dramatic, whereas the other two polychaete species undergo major morphological alterations. For instance, in *H. elegans*, the gut forms very early in the larval stage with no circumferential expansion of the segmental tissue. Specific protein spots may be required for gut formation in the early larval phase. Competent *P. vexillosa *larvae accumulate neurochaetes, sensory structures, and feeding structures late in larval life as they undergo transient structural reorganization [20] whereas *Capitella *sp. I larvae metamorphose spontaneously and require little tissue remodulation, such as elongation of the body and loss of cilia leading to minor morphological changes [7].

Notably, phosphoprotein down-regulation (>100 spots) was also drastic during development from the competent stage to the adult stages in *P. vexillosa *[13] and *H. elegans *[14]. In contrast, only ninteen phosphoprotein spots were down-regulated in *Capitella *sp. I, indicating that no drastic change occurred. This observation supports our hypothesis that the phosphoprotein expression pattern during larval metamorphosis in *Capitella *sp. I is relatively different from that of other polycheate species [17,18]. In general, marine invertebrate larvae have evolved to undergo speedy metamorphosis to minimize the time that they are most vulnerable to predation [21,22]. *P. vexillosa *complete their settlement and metamorphosis processes within three hours after attaining competency, whereas the initial phase of this process is finished in as little as 10 min in competent larvae of *H. elegans*, while metamorphosis is achieved 11 to 12 hours post-settlement[23].

In this study, we detected many phosphoproteins in low abundance in both the COM and JUV stages (Figure 4 and 5, spots 1-16). Phosphorylation of these proteins appears to be necessary for *Capitella *sp. I to undergo transient structural re-organization, including elongation of body segments, production of hooded hooks, and preparation of muscle tissues and organs during metamorphosis. These spots with low abundance were not identified because of difficulties in obtaining satisfactory protein identification by LC-MS. Identification of all differentially expressed phosphoproteins in the future may help to explain the possible role of protein phosphorylation during the transition process from larvae to juveniles in polychaetes.

Metamorphosis in many marine invertebrates typically involves the loss of larval characteristics mediated by protein degradation and various forms of programmed cell death [24,25]. Abundant expression of tubulins in COM larvae and the subsequent down-regulation in the JUV may be related to larval tissue degeneration and cellular disorganization during metamorphosis. Tubulin isoforms have also been found to be down-regulated during larval development of many invertebrates such as *H. elegans *[13] and *P. vexillosa *[14]. Tubulin forms the core structure of the cilia and contributes to the ciliation of all components of the "opposed-band feeding system" in polychaetes. Tubulins are also the building blocks of microtubules and play important roles in many cellular processes such as cell division and cell migration [26]. The spatio-temporal expression of different tubulin isotypes may be related to a variety of physiological functions and post-translational modifications [27,28]. TP is an actin-binding protein that regulates the actin mechanism in muscle contractions by responding to an intracellular rise in Ca^2+ ^levels [29]. The up-regulation of TP may be related to muscle contractions during body elongation and preparation of new muscle tissues required for juveniles. These results suggest that cytoskeletal dynamics occur more frequently when larvae are reaching competency to metamorphose.

Among other identified proteins, ICH, EPI, ATP2, and Vtg are involved in the citric acid cycle, glycolysis, and energy metabolism. During competency and early metamorphosis, a worm's metabolism becomes highly active as the larvae require extra energy supplies to initiate and fuel the transition from larva to juvenile. EP1 is a bifunctional enzyme from the hydrolase superfamily that is mainly involved in the amino-acid biosynthesis pathway [30]. EP1 is also up-regulated and phosphorylated in the competent larvae of *P. vexillosa *[31]. ICH and EP1 may be important for the activation of the entire energy-producing pathway during the competent stage [32,33]. ATP2 is a ubiquitous mitochondrial enzyme that plays a key role in biological energy metabolism [34]. In *Capitella *sp. I, we found that the expression of ATP2 increased in the JUV stage. Because it has been found that several energy metabolism pathways act complementarily as a temporal energy buffer under specific conditions in a variety of biological systems [35], we suggest that the differential regulation of ATP synthase during metamorphosis in *Capitella *sp. I results from the changes in the oxidative energy metabolism in the mitochondria. Abundant expression of Vtg in the COM may serve as a source of energy during development, particularly in larvae that are lecithrotrophic and do not feed throughout metamorphosis [36]. In addition, this protein is involved in the ion and molecule transporting process [37] and plays a role in the synthesis of the brooding tube, longevity, and the immune system [38].

Many environmental, chemical, and physical stressors influence larval development and metamorphosis of marine invertebrates [39]. Three of the identified proteins, HSP90, TH, and TPx, which are often involved in coping with oxidative stress, were up-regulated in the COM. One possible explanation for this is that non-feeding competent polychaetes continuously engage in the degeneration of larval structures and the search for habitats, leading to an increase in oxidative stress [40]. To counter balance the stress, a series of protective responses is triggered in the larvae. Abundant expression of TH and HSP90 might be required to confer protection under stressful conditions [41]. TH and TPx are involved in important cellular processes, such as cell-cycle control, apoptosis, and stress response [42,43]. HSP 90 acts as the 'translator' to transduce environmental changes in cell signaling pathways [44-46] and its resistance to stress can be achieved by a highly conserved and functionally interactive network of chaperone proteins that can rapidly respond to environmental stresses [47]. TAF is involved in transcription and translation processes related to a variety of key development pathways [48,49]. Up-regulation of TAF at both protein and transcription levels in juveniles may indicate cell signaling and transcriptional regulation of juvenile tissue differentiation from the arrested larval rudiments.

Interestingly, we identified three abundant hypothetical proteins whose expression was down-regulated in the JUV. Although the function of these "conserved" proteins has not been completely identified, they probably play an important role in *Capitella sp *I larval metamorphosis. The identification and elucidation of the function of these hypothetical proteins is further required for understanding the molecular processes associated with larval metamorphosis.

### Comparison of proteome profiles with non-polychaete species

In general, metamorphosis in marine invertebrates typically involves the breakdown of larval tissues followed by the emergence of juvenile structures [21], but different larval species may differ in attaining competency and metamorphosis transitions [50]. Our previous studies revealed a substantial reduction in the number of proteins spots during metamorphosis in *B. neritina *and *B. amphitrite *[12,51]. On the contrary, the reduction in proteins spots is not substantial in *Capitella sp *I. These obvious differences in proteome changes between these species can be accounted for morphological changes during larval metamorphosis. For instance, *B. neritina *and *Ba. amphitrite *initiate larval metamorphosis after attachment and their metamorphosis into juveniles is a rapid process involving substantial morphological changes [52,51]. *B. neritina *larvae settle immediately after being released from brooding adults. The settling larvae of *Ba. amphitrite *display site-selection behavior. In this study, we found abundant expression of tubulin isoforms in the COM larvae and subsequent down-regulation in the JUV. In comparison, tubulin isoforms have also been found to be down-regulated during larval development of *B. neritina *and *B. amphitrite *[12,51]. Similarly, the up-regulation of TP both in *Capitella sp *I and *B. neritina *may be related to muscle contractions during body elongation and preparation of new muscle tissues. In the abalone *Haliotis rufescens*, degeneration and differentiation of muscles during the metamorphic transition are regulated by divergent forms of tropomyosin [53]. The down-regulation of Vtg in these species may serve as a source of energy for the non-feeding swimming larvae [37]. Similarly, we also observed the down-regulation of oxidative stress proteins such as HSP90, TH in the juvenile stages of *B. neritina and B. amphitrite*, indicating oxidative stress in the marine invertibrate larvae [46,47]. ICH participates in the citric acid cycle. Evidence has been presented that it is phosphorylated in the competent larvae of *B. amphitrite and B. neritina *[51].

## Conclusion

In this study, we have reported changes in expression levels of both proteins and phosphoproteins in two developmental stages during larval metamorphosis in *Capitella *sp. I. Twenty-three differentially expressed proteins during larval metamorphosis were identified. Cytoskeletal proteins, oxidative stress proteins, and energy metabolism proteins appeared to be directly involved in the larval metamorphosis process. Subsequent studies on differential expression of some of the selected proteins at the translational and transcriptional levels supported our proteomics-based results. This is the first proteomic study to examine changes in the protein expression level during larval metamorphosis in the polychaete *Capitella *sp. I. It is a starting point for further investigation into the functions of the identified proteins.

## 4. Methods

### 4.1. Larval culture and sample collection

Competent larvae (COM) and juveniles (JUV) of *Capitella *sp. I (Figure 1) were obtained from adults using the process described by Cohen and Pechenik [54] with minor modifications. Briefly, adult colonies were collected from the sediment of a mudflat in Hong Kong (22.4167° N and 114.2667° E) and maintained in the laboratory in Pyrex glass beakers in a seawater table at 25°C and 34 ppt salinity. Cultures were periodically sub-cultured with sediment obtained from mudflats near the Hong Kong University of Science and Technology, Hong Kong and supplemented with Tetra-Marine, a commercial fish diet, as a food source. Brooding females were isolated and placed in clean dishes. Fifty to 300 larvae per brood were collected and were transferred into lysis buffer immediately after being released from their brooding tubes (7 M of urea, 2 M of thiourea, 4% CHAPS, 1% DTT, protease and phosphatase inhibitors) and frozen at -80°C. Larvae obtained from 10 to 20 broods were pooled. A thin layer of finely (<80 μm) sieved sediment was added to each container to induce larval metamorphosis. Juveniles, indicated by the loss of both prototrochal and telotrochal ciliary bands, were obtained from several broods (about 20 to 30) and collected within 24 h after the larvae were added to the containers. Juveniles were preserved in the same way as the competent larvae after collection and kept frozen at -80°C until further analysis. Three biologically independent replicates were used for the proteomic analysis.

### 4.2. Preparation of protein samples and two-dimensional gel electrophoresis

Sample preparation was carried out as described by Mok *et al*. [13] with slight modifications. Briefly, the competent larvae and juveniles were transferred to a lysis buffer and sonicated on ice using ten 5 sec blasts of 15% amplitude with 10 sec pauses between blasts. The samples were then centrifuged at 13,000 rpm for 10 min, and the proteins in the supernatant were purified using a two-dimensional gel electrophoresis (2-DE) cleanup kit (Bio-Rad, Hercules, CA, USA). The purified protein pellets were resolubilized in lysis buffer and the protein concentration was determined using a modified Bradford method [55]_. _Three-hundred micrograms of each protein sample were sonicated for 10 min and incubated at room temperature for 2 hr to enhance protein solubilization. Rehydration was carried out using 300 μl of the sample in a rehydration buffer (7 M of urea, 2 M of thiourea, 4% CHAPS, 40 mM of dithiothreitol (DTT), 0.5% pI 4-7 ampholyte, and 1% bromophenol blue) on 17 cm immobilized pH gradient (IPG) strips (pH 4-7) for ~16 hr. The samples were then subjected to isoelectrical focusing (IEF) using a Protean IEF Cell (Bio-Rad, Hercules, CA, USA). Focusing was carried out at 250 V for 20 min and then along a gradient from 1,000 to 8,500 V for 2 hr to give a total of 60,000 Vh. The current did not exceed 50 mA per strip. After IEF, reduction and alkylation of the IPG strips were carried out using DTT and iodoacetamide (IAA), and two-dimensional SDS-PAGE was performed following the protocol described by Zhang *et al. *[14].

### 4.3. Multiplex fluorescent gel staining and image analysis

The 2-DE gels were fixed overnight in 40% methanol and 10% acetic acid and sequentially stained for phosphoproteins with Pro-Q Diamond (Invitrogen, CA, USA) and for total proteins with Sypro Ruby (Invitrogen, CA, USA) according to the manufacturer's instructions with minor modifications. To stain the phosphoproteins, the 2-DE gels were incubated for 3 hr in ProQ Diamond, followed by destaining with 20% acetonitrile (ACN) in 50 mM of sodium acetate (pH 4.0) for 3 hr. After destaining, the gels were scanned for phosphoprotein spots using a Typhoon trio imager (GE Healthcare, Piscataway, NJ, USA) at an excitation of 532 nm with a 610 BP 30 emission filter. After the scan images were acquired, the gels were incubated overnight in the dark with Sypro Ruby for total protein detection. They were again scanned using the Typhoon trio imager at an excitation of 582 nm with a 610 BP 30 emission filter. The gels were destained in 10% methanol and 7% acetic acid for 1 hr. To prepare for protein spot excision and subsequent mass spectrometry (MS) analysis, the gels were stained with the modified G-250 Colloidal Coomassie Blue (CCB). Three replicate gels stained with phosphoprotein stain and total protein stain were grouped accordingly and compared. Quantitative and qualitative analysis were carried out using the PDQuest software (Bio-Rad, Hercules, CA, USA) as described by Thiyagarajan *et al. *[51]. The spot intensities were normalized so that the total density of each image was equal. Only spots that were present in all three replicate gels were considered. A 1.5-fold threshold was set for quantitative detection of protein changes between two developmental stages. Protein and phosphoprotein spots that were significantly different (Student's *t*-test, *p *< 0.01) in successive stages were considered to be up- or down-regulated.

### 4.4. Mass spectrometry

Forty abundant and differentially expressed protein spots were subjected to MS analysis. Each protein was manually inspected for differential expression in order to match the PDQuest spot detection and to ensure that selected spots were reproducibly detected among three replicates. The protein spots were excised, washed, and digested in 20 μL of 12.5 ng/mL trypsin (Promega, Madison, WI, USA) in 10% acetonitrile and 10 mM of NH_4_HCO_3 _at 37°C for 16 hr. The peptides were extracted and dried in a speed vacuum following the protocol described by Qian *et al. *[56]. The peptides were dissolved in 3 μL of 0.1% trifluoroacetic acid (TFA) and 3 μL of each sample was spotted on an AnchorChip PAC 384 HCCA (Bruker Daltonics, Bellirica, MA, USA) target plate pre-coated with a matrix of cyano-4-hydroxy-cinnamic acid, followed by desalting with 10 mM of ammonium phosphate in 0/1% TFA. The samples were analyzed using an Autoflex III TOF/TOF mass spectrometer (Bruker Daltonics, Bellirica, MA, USA) as described by Lu *et al. *[57]. External calibration was performed using Bruker peptide calibration standards. Mass spectra (MH+) were acquired by the FlexControl software (version 3.0, Bruker Daltonics), which recorded in the range of 800-3500 Da. The MS/MS information was obtained in the LIFT (laser-induced forward transfer) mode. The MS and MS/MS spectra were combined using the BioTools software (version 3.1, Bruker Daltonics) and searched against the *Capitella *genome database (*Capitella capitata *v 1.0 Filtered Gene Modules) using the MASCOT software (Matrix science). Due to incompleteness of the *Capitella *sp. I genome database, the MS spectra were also searched against NCBI nr database to confirm that the proteins identified by the *Capitella *sp. I database were correct. The search parameters were set at 50 ppm for peptide tolerance and 0.2 Da for the MS/MS tolerance. Protein scores greater than 58 and 81 were considered statistically significant (*p *< 0.05) for the *Capitella *sp. I and NCBI nr databases, respectively. Search results from the combined spectra that were statistically significant (*p *< 0.05) were accepted.

### 4.5. Western-blot analysis

Western blot analysis was performed in three independent biological replicates to confirm the differential expression of the HSP90 and tyrosine 3-monooxygenase proteins following the protocol described by Zhang *et al *[58]. Briefly, 20 μg of protein lysates from competent larvae and juveniles used in the same 2-DE experiment were separated on 10% SDS-PAGE and transferred to an Immobilon transfer membrane (Millipore, MA, USA). Blots were probed with 1:1000 diluted monoclonal antibodies of anti-HSP90 (Cell Signaling, Danvers, USA) and anti-tyrosine 3-monooxygenase (Abcam, Cambridge, USA) by incubating for ~16 h in 4°C. The membranes were then probed with horseradish peroxidase-conjugated secondary antibodies (1:5000 dilution) for 1 hr, followed by chemiluminescent detection using an ECL Western blotting analysis system (Millipore, Billerica, MA).

### 4.6. Semi-quantitative real-time PCR

To validate the results of the differential proteome analysis at the mRNA level, quantitative real-time PCR (qRT-PCR) was performed following the protocol detailed by Wong *et al. *[59]. Briefly, total RNA from the competent larvae and juveniles was isolated using the TRIzol reagent (Invitrogen, CA, USA), according to the supplier's instructions. The extracted total RNA was digested with DNase (Turbo DNA-free™ Kit, Applied Biosystems, CA, USA) to remove trace DNA contaminants. The cDNA was synthesized from 2 μg of total RNA from each stage using M-MLV reverse transcriptase (USB, Cleveland, OH, USA) with random hexamer primer. Gene-specific primers were designed based on the nucleotide sequence of the target protein in the *Capitella capitata *genome database. The *Capitella *sp. I 18S RNA gene (GenBank Accession No. AF508118) was chosen as the reference gene for normalizing the expression levels of the target genes. qRT-PCR assays for each target gene were performed in triplicate and repeated twice. All qRT-PCR assays were carried out using iTaq SYBR Green Supermix with ROX (BioRad, USA) and run on the Stratagene mx3000p PCR machine (Agilent Technologies, Santa Clara, CA, USA). The qRT-PCR data were analyzed by the 2-ΔΔCT method as described by Livak and Schmittgen [60].

## Abbreviations

COM: competent larvae; JUV: juveniles; TUB: tubulin; ACT: actin; Vtg: vitellogenin; ATP2: ATP synthase; TM: tropomyosin; ICH: isocitrate dehydrogenase-2: EP1: enolase-phosphatase; TPx: thioredoxin peroxidase; HSP90: heat shock protein 90; TAP: template activating factor; HP: hypothetical protein; PKC: protein kinase C; JH: juvenile hormone

## Competing interests

The authors declare that they have no competing interests.

## Authors' contributions

PYQ conceptualized the study and revised the manuscript; KHC prepared the samples, performed the 2DE, protein enrichment, MALDI-TOF MS analysis, Western blot, RT-PCR and drafted the initial version of the manuscript; LS carried out the larval culture and collection and participated in the sample preparation. All of the authors read and approved the final version of the manuscript.
